# Impaction bone grafting of the acetabulum at hip revision using a mix of bone chips and a biphasic porous ceramic bone graft substitute: Good outcome in 43 patients followed for a mean of 2 years

**DOI:** 10.3109/17453670902884767

**Published:** 2009-04-01

**Authors:** Ashley W Blom, Vikki Wylde, Christine Livesey, Michael R Whitehouse, Steve Eastaugh-Waring, Gordon C Bannister, Ian D Learmonth

**Affiliations:** ^1^Department of Academic Orthopaedics, University of Bristol, BIRC Research Labs, Avon Orthopaedic CentreBristol, BS10 5NB; ^2^Department of Orthopaedics, North Bristol NHS Trust, Avon Orthopaedic Centre, Southmead HospitalBristol, BS10 5NB

## Abstract

**Background and purpose** One of the greatest problems of revision hip arthroplasty is dealing with lost bone stock. Good results have been obtained with impaction grafting of allograft bone. However, there have been problems of infection, reproducibility, antigenicity, stability, availability of bone, and cost. Thus, alternatives to allograft have been sought. BoneSave is a biphasic porous ceramic specifically designed for use in impaction grafting. BoneSave is 80% tricalcium phosphate and 20% hydroxyapatite. Previous in vitro and in vivo studies have yielded good results using mixtures of allograft and BoneSave, when compared with allograft alone. This study is the first reported human clinical trial of BoneSave in impaction grafting.

**Methods** We performed a single-institution, multi-surgeon, prospective cohort study. 43 consecutive patients underwent revision hip arthroplasty using BoneSave and allograft to restore missing bone in the acetabulum. 9 patients had cemented acetabular components implanted and 34 uncemented. 10 patients had cemented femoral components implanted and 1 had an uncemented femoral component. 32 patients did not have their femoral component revised.

**Results** No patients were lost to follow-up. At a mean follow-up of 24 (11–48) months, there were no re-revisions and there was no implant migration. 1 acetabular component had confluent lucent lines at the implant-graft interface. Complications were rare (1 fracture, 2 dislocations). Patient satisfaction with the procedure was high.

**Interpretation** Short-term results indicate that impaction grafting of BoneSave and allograft is an effective method of dealing with loss of bone stock at revision hip surgery.

## Introduction

Dealing with loss of bone stock is one of the greatest challenges facing the revision hip surgeon. Initial attempts to address this problem yielded poor results. The Exeter and Nijmegen groups popularized impaction grafting of donor allograft in the early 1990s (Gie et al. 1993, Slooff et al. 1996). Their excellent clinical results have not always been reproduced elsewhere. Eldridge et al. (1997) reported an early massive subsidence of 12% of femoral stems. In addition, allografts in general have been associated with problems of infection (Huo et al. 1992, Simonds et al. 1992), antigenicity (Friedlander et al. 1984), availability (Galea et al. 1998), and cost (Tomford et al. 1981).

For those reasons, a number of alternatives to allograft have been investigated. These include xenografts, various ceramics such as hydroxyapatite and other calcium-phosphates, coral, bamboo, and reinforced collagen matrices (Itokazu et al. 1996, Jensen et al. 1996, Li et al. 1997). Initial results have identified certain shortcomings with both xenografts and glass-ionomers. Xenografts osseointegrate less well than allograft, and, as with allograft, they show variability in particle size, particle morphology, and impaction properties. They are also associated with problems of infection (Charalambides et al. 2005) and antigenicity (Begley et al. 1995).

Glass-ionomers are non-compressible and, as they are non-porous, they allow only peripheral osseointegration with no effective osseoconduction within the ceramic particles. Tsuruga et al. (1997) and Kuhne et al. (1994) have shown that a pore size of approximately 300–400 µm in ceramics will allow optimal osseoconduction.

Hydroxyapatite and tricalcium phosphate ceramics have been shown to osseointegrate (Itokazu et al. 1996, Ransford et al. 1998), but concerns have been raised as to their ability to maintain their structural integrity under load (Hanft et al. 1995).

BoneSave is a biphasic porous ceramic bone graft substitute, specifically designed for use in impaction grafting in revision hip surgery. It is sintered at a high temperature in order to give it the requisite compressive strength to withstand the high compressive and shear forces associated with impaction grafting. It has been tested extensively both in vitro (Blom et al. 2002, Arts et al. 2005a, Van Haaren et al. 2005) and in vivo (Arts et al. 2005b, Blom et al. 2005a), but as yet there have been no published human clinical reports. Despite this, it is used throughout the world and in over 175 hospitals in the UK (Stryker, Newbury, UK, personal communication).

We report the first human cohort study using BoneSave to restore missing bone at revision hip surgery.

## Patients and methods

From 2003 through 2006, 43 hips in 43 patients at our hospital underwent revision total hip replacement for aseptic loosening with contained bony defects. All bony defects were grafted with a 50:50 volume mix of BoneSave (Table 1) and allograft from donor human femoral heads.

**Table 1.** Characteristics of BoneSave

Composition	80% tricalcium phosphate
	20% hydroxyapatite
Sintering temperature	> 1,200°C (to achieve optimum hardness)
Crystallinity	High (> 80%)
Porosity	50% by volume
	Pore size 300–500 µm
Granule size	2–8 mm

The inclusion criterion was: all patients undergoing revision hip surgery for aseptic loosening with contained acetabular defects.

The patients were a consecutive series consisting of all patients who received BoneSave at the Avon Orthopaedic Centre, and the operating surgeons were the 4 authors (AB, GB, SEW and IL)

### Patient demographics

27 patients were female and 16 were male. Mean age was 71 (42–90) years. The mean follow-up was 24 (11–48) months, with a total of 78 person years of observations. At 11 months, there were 43 patients at risk (i.e. still undergoing follow-up) and at 12 months there were 39 patients at risk. This fell to 18 patients at 24 months, 6 patients at 36 months, and 2 patients at 48 months. 9 patients had had cemented acetabular components and 34 had had uncemented acetabular components. 11 patients had contained femoral defects that were grafted. 10 patients had cemented femoral components implanted and 1 had an uncemented femoral component implanted. The remaining 32 patients did not have the femoral component revised.

The graft material was a 50:50 by volume mix of morsellized allograft and BoneSave. Allograft was harvested from donor femoral heads and milled in a bone mill. Impaction grafting was performed as described by Bolder et al. (2007) using metal impactors and a hammer to compress graft into the acetabular defect. As the defects were contained, it was not necessary to use meshes or cages. Frozen sections and cultures were not taken to exclude infection. The uncemented cups were Pinnacle (De Puy International, Leeds, UK) or Procotyl (Wright, Arlington, TN) and the cemented were OGEE LPW (De Puy International). All patients were mobilized to full weight bearing within 48 h of surgery.

Patients were then followed up radiographically and clinically at 3 months, 6 months, 1 year, and then annually. At latest review, they were sent a postal questionnaire consisting of the Oxford hip score (Dawson et al. 1996), the SF-12 (Ware et al. 1996) and the Satisfaction Scale for Joint Replacement Arthroplasty (Mahomed et al. 1997). The Oxford hip score is a 12-item patient-reported outcome measure assessing hip pain and functional limitations on a scale of 12–60 (low-high disability). The SF-12 is a general health questionnaire, which produces a Physical Component score and Mental Component score scale from 0 to 100 (representing poor to excellent health). The Satisfaction Scale for Joint Replacement Arthroplasty consists of 4 questions asking respondents to rate their satisfaction with hip replacement in terms of pain relief, ability to perform activities of daily living (ADLs), ability to partake in leisure activities, and overall satisfaction. A total satisfaction score can then be calculated on a scale of 0–100 (ranging from full dissatisfaction to full dissatisfaction). In addition, a question was included asking patients if they would undergo the operation again, based on their experience of the hip replacement.

## Results

No new technical problems were identified with the impaction process. The operating surgeons were all experienced with the impaction technique using allograft, and found that impaction of the mixture to be no more technically demanding. There were no revisions and no impending revisions of the construct at 11–48 months. There were no infections. There were 2 single dislocations, one on day 2 and the other 6 weeks postoperatively. Both dislocations were reduced closed. There was 1 femoral fracture distal to the tip of the prosthesis, which occurred 4 months after surgery and was fixed with a strut graft and femoral plate. The patient made an uneventful recovery.

No patients were lost to follow-up. 38 of 43 questionnaires were returned; 4 patients died and 1 patient suffered from advanced dementia, and was thus unable to complete the questionnaire. Taking missing data into account, Oxford hip scores were available from 32 patients, SF-12 scores from 35 patients, satisfaction scores from 35 patients, and the answer on repeating the operation from 34 patients.

The mean overall satisfaction score was 75 (range 17–100; SD 27; 95% CI: 66–84) (Table 2).

**Table 2.** Satisfaction scale

Satisfaction	Very satisfied	Somewhat satisfied	Somewhat dissatisfied	Very dissatisfied
overall	24	7	3	1
with pain relief	22	8	3	2
with ability to perform ADLs	14	13	3	5
with leisure activities	12	13	7	3

The mean Oxford hip score was 26.9 (range 13–55; SD 11; 95% CI: 23–31) (Table 3).

**Table 3.** Oxford hip score

Oxford hip score	12–20	21–30	31–40	41–50	51–60
No. of patients	10	11	6	4	1

The mean Mental Component score was 48 (range 20–66; SD 11; 95% CI: 31–39) and the mean Physical Component score was 36 (range 21–57; SD 11; 95% CI: 44–52).

27 patients stated that they would go through the operation again, 4 stated they would not have the operation again, and 3 patients were unsure.

### Radiographic results

All radiographs were available for assessment. No radiographs showed any signs of component migration, as measured on plain radiographs referencing from the tear drop and the transischial line. BoneSave granules were not visible in the soft tissues on postoperative radiographs, but were clearly visible in the impaction grafting construct. There were no incidences of heterotopic ossification or accelerated polyethylene wear, as assessed by measuring femoral head concentricity. 1 radiograph showed confluent lucent lines of greater than 2 mm thickness around the acetabular component. At 3-year follow-up, this patient had an excellent clinical result and was pain-free with a normal gait (see Figures 1A and 1B). Case 2 (illustrated in Figures 2A, 2B, and 2C) was typical of our series. Of the patients who received an uncemented component, 10 of 34 had lucent lines at the component-graft interface, which were all less than 2 mm in thickness at latest review. 4 of these were in DeLee and Charnley zones 1, 2, and 3, 4 were in zone 3 only, and 2 were in zones 1 and 2. Of the patients who received a cemented component, 1 of 9 had a lucent line at the cement-graft interface, which was less than 2 mm in thickness at latest review and was present in DeLee and Charnley zones 1 and 2.

**Figure 1A.**
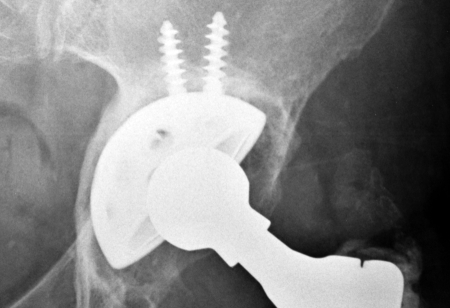
1 year postoperatively. Demonstrates 2-mm-thick confluent radiolucent line around the acetabular component.

**Figure 1B.**
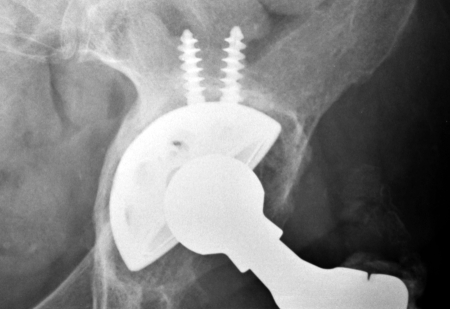
3 years postoperatively, showing stable radiolucency with excellent clinical results.

**Figure 2A.**
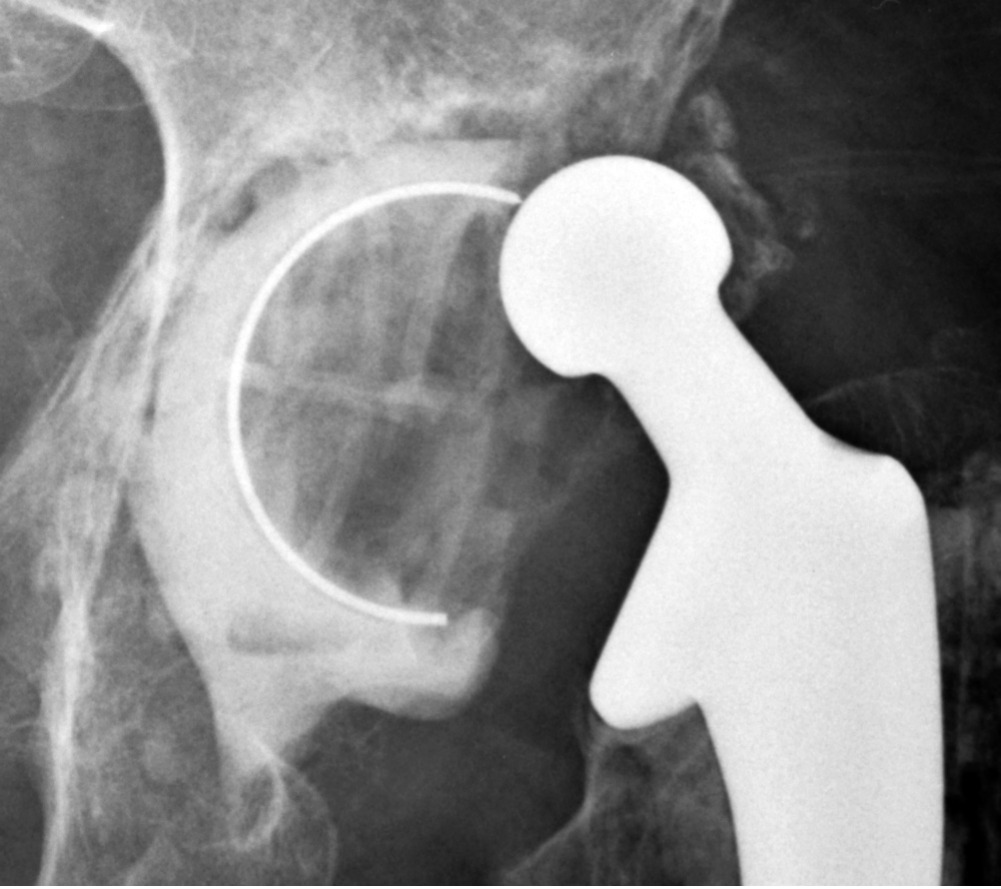
Preoperatively. Loose acetabular component and migration, leading to dislocation.

**Figure 2B.**
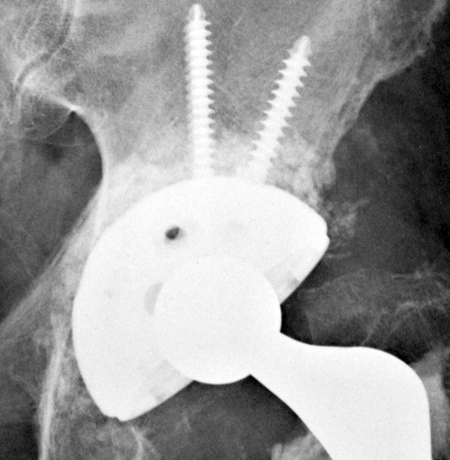
Postoperatively. Well-fixed acetabular component with visible BoneSave granules impacted.

**Figure 2C.**
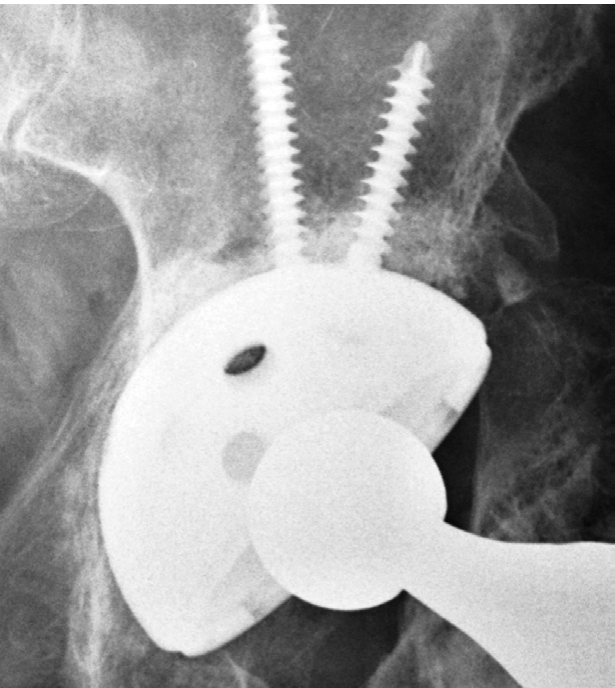
3 years postoperatively. The acetabular component remains well fixed with integration of BoneSave graft.

## Discussion

Our results are short-term and must be interpreted with caution. It remains to be seen whether they may reflect long-term success. A number of centers have reported excellent results with impaction grafting of all allograft in the acetabulum, in the medium to long term (Schreurs et al. 2004, Deakin and Bannister 2007, Somford et al. 2008). Not all results of impaction grafting of the acetabulum with allograft have been favorable, however, and there may be bulk limitations (Van Haaren et al. 2007).

The bone defects in our patients are simply described as being contained rather than being classified according to one of the commonly used classification systems such as the Paprosky (Paprosky et al. 1994) or AAOS (D'Antonio 1992). Previously published work from our institution has demonstrated that these classification systems lack inter- and intraobserver reliability, and do not correlate very well with intraoperative findings (Gozzard et al. 2003).

Our study has a number of shortcomings, in that it was not a randomized controlled trial, in that the follow-up was short, and that both cemented and uncemented acetabular components were used. Furthermore, the method of assessing component migration was not very sensitive compared to methods such as RSA; thus, small migrations may have been missed. The results do, however, demonstrate that the concept works in the short term and they show the need for a randomized, controlled trial comparing impaction grafting with allograft alone with mixtures of allograft and bone-graft substitutes.

The particular biphasic porous ceramic bone graft substitute we used has been shown to be both more stable and more reproducible than allograft in mechanical engineering testing with both femoral (Blom et al. 2002) and acetabular models (Arts et al. 2005a). Furthermore, ovine and caprine studies have shown impaction grafting with BoneSave to be as least as good as allograft in the clinical setting with regard to function, radiographic analysis, and new bone formation (Arts et al. 2005b, Blom et al. 2005a). As the ceramic was mixed with allograft, it would not completely eliminate the risks of antigenicity and infection. However, using a ceramic bone graft substitute in this way markedly reduces the amount of allograft bone implanted and thereby reduces the risk of immune reaction and infection.

We found none of the adverse reactions associated with other types of bone graft substitutes. Previous studies with Surgibone (a bovine-derived bone graft substitute) have shown an extremely high revision rate for presumed infection (Charalambides et al. 2005). In contrast, there were no infections in this study.

BoneSave has a number of advantages over allograft, in that it is reproducible, provides a stable construct (with no implant migration), osseointegrates (Blom et al. 2005a), and has no intrinsic risk of infection and antigenicity. In addition, it has a pore size that has been demonstrated to be optimal for osseoconduction (Tsuruga et al. 1997). Because BoneSave was mixed with allograft, it reduced the amount of allograft needed and thereby reduced the risk of infection and antigenicity.

We found no untoward side effects, such as unexpected heterotopic ossification. The complication rates were low compared to other studies of revision hip surgery (Eldridge et al. 1997, Blom et al. 2003, 2005b).

The patients were satisfied with the clinical outcome, particularly with regard to pain relief, and most of the patients stated that they would have undergone the same procedure again.

The high percentage of fine lucent lines evident at the latest radiographic review may be a predictor of future implant migration, and highlights the need for long-term review. However, with impaction grafting, when catastrophic failure has occurred (be it because of infection or implant migration), it has usually occurred within 1 year of surgery (Charalambides et al. 2005, Eldridge et al. 1997). Despite the fact that BoneSave appears to be safe according to our evaluation, longer follow-up is necessary before this material can be recommended for general use.

## References

[CIT0001] Arts JJ, Schreurs BW, Buma P, Verdonschot N (2005a). Cemented cup stability during lever-out testing after acetabular bone impaction grafting with bone graft substitutes mixes containing morselized cancellous bone and tricalcium phosphate-hydroxyapatite granules.. Proc Inst Mech Eng [H]..

[CIT0002] Arts JJ, Gardeniers JW, Welten ML, Verdonschot N, Schreurs BW, Buma P (2005b). No negative effects of bone impaction grafting with bone and ceramic mixtures.. Clin Orthop.

[CIT0003] Begley C, Doherty M, Mollan R, Wilson D (1995). Comparative study of the osteoinductive properties of bioceramic, coral and processed bone grafts.. Biomaterials.

[CIT0004] Blom AW, Grimm B, Miles AW, Cunningham J, Learmonth ID (2002). Subsidence in impaction grafting. The effect of adding a ceramic bone graft substitute. Engineering in Medicine.. Proc Inst Mech Eng [H].

[CIT0005] Blom AW, Taylor AH, Pattison G, Whitehouse S, Bannister GC (2003). Infection after total hip arthroplasty. The Avon experience.. J Bone Joint Surg (Br).

[CIT0006] Blom AW, Cunningham J, Hughes G, Lawes TJ, Smith N, Blunn G, Learmonth ID, Goodship AE (2005a). Functional and biological compatibility of ceramic bone graft substitutes as allograft extenders for use in impaction grafting of the femur.. J Bone Joint Surg (Br).

[CIT0007] Blom AW, Astle L, Loveridge J, Learmonth I (2005b). Acetabular liner revision has a high risk of dislocation.. J Bone Joint Surg (Br).

[CIT0008] Bolder SBT, Verdonschot N, Schreurs BW (2007). Technical factors affecting cup stability in bone impaction grafting.. Proc Inst Mech Eng [H]..

[CIT0009] Charalambides C, Beer M, Cobb AG (2005). Poor results after augmenting autograft with xenograft (Surgibone) in hip revision surgery: a report of 27 cases.. Acta Orthop..

[CIT0010] D'antonio JA (1992). Periprosthetic bone loss of the acetabulum: classification and management.. Orthop Clin North Am.

[CIT0011] Dawson J, Fitzpatrick R, Carr A, Murray D (1996). Questionnaire on the perceptions of patients about total hip replacement.. J Bone Joint Surg (Br).

[CIT0012] Deakin DE, Bannister GC (2007). Graft incorporation after acetabular and femoral impaction grafting with washed irradiated allograft and autologous marrow.. J Arthroplasty.

[CIT0013] Eldridge JDJ, Smith EJ, Hubble MJ, Learmonth ID (1997). Massive subsidence following femoral impaction grafting.. J Arthroplasty.

[CIT0014] Friedlander GE, Strong DM, Sell KW (1984). Studies on the antigenicity of bone. Donor specific anti-HLA antibodies in human recipients of freeze-dried allografts.. J Bone Joint Surg (Am).

[CIT0015] Galea G, Kopman D, Graham BJM (1998). Supply and demand of bone allograft for revision hip surgery in Scotland.. J Bone and Joint Surg (Br).

[CIT0016] Gie GA, Linder L, Ling R, Simon J-P, Sloof TJ, Timperley A (1993). Impacted cancellous allografts and cement for revision total hip arthroplasty.. J Bone Joint Surg (Br).

[CIT0017] Gozzard C, Blom A, Taylor A, Smith E, Learmonth I (2003). A comparison of the validity and reliability of bone stock loss classification systems used for revision hip surgery.. J Arthroplasty.

[CIT0018] Hanft J, Sprinkle R, Surprenant M, Werd M (1995). Implantable bone substitute materials.. Implant Biomat.

[CIT0019] Huo MH, Friedlander GE, Salvati EA (1992). Bone graft and total hip arthroplasty. A review.. J Arthroplasty.

[CIT0020] Itokazu M, Matsunaga T, Ishii M, Kusakabe H, Wyni Y (1996). Use of arthroscopy and interporous hydroxyapatite as a bone graft substitute in tibial plateau fractures.. Arch Orthop Trauma Surg.

[CIT0021] Jensen S, Aaboe M, Pinholt E, Hjorting-hansen E, Melsen F, Ruyter E (1996). Tissue reaction and material characteristics of four bone substitutes.. Int J Oral Maxillofac Implants.

[CIT0022] Kuhne J-H, Bartl R, Frisch B, Hammer C, Jannson V, Zimmer M (1994). Bone formation in coralline hydroxyapatite: effects of pore size studied in rabbits.. Acta Orthop Scand.

[CIT0023] Li SH, Liu Q, Dewijn JR, Zhou BL, Degroot K (1997). In vitro calcium phosphate formation on a natural composite material, bamboo.. Biomaterials.

[CIT0024] Mahomed N, Sledge CB, Daltroy L, Fossel A, Katz J (1997). Self-administered satisfaction scale for joint replacement arthroplasty.. J Bone Joint Surg (Br).

[CIT0025] Paprosky WG, Perona PG, Lawrence JM (1994). Acetabular defect classification and surgical reconstruction in revision arthroplasty. A 6-year follow-up evaluation.. J Arthroplasty.

[CIT0026] Ransford A, Morley T, Edgar M (1998). Synthetic porous ceramic compared with autograft in scoliosis surgery.. J Bone Joint Surg (Br).

[CIT0027] Schreurs BW, Busch VJ, Welten ML, Verdonschot N, Slooff TJ, Gardeniers JW (2004). Acetabular reconstruction with impaction bone-grafting and a cemented cup in patients younger than fifty years old.. J Bone Joint Surg (Am).

[CIT0028] Simonds RJ, Holmberg SD, Hurwitz RL (1992). Transmission of human immunodeficiency virus type 1 from a seronegative organ and tissue donor.. N Engl J Med.

[CIT0029] Slooff TJ, Buma P, Schreurs BW, Schimmel JW, Huiskes R, Gardeniers J (1996). Acetabular and femoral reconstruction with impacted graft and cement.. Clin Orthop.

[CIT0030] Somford MP, Bolder SBT, Gardeniers JW, Slooff TJ, Schreurs BW (2008). Favorable survival of acetabular reconstruction with bone impaction grafting in dysplastic hips.. Clin Orthop.

[CIT0031] Tomford W, Starkweather R, Goldman M (1981). A study of the clinical incidence of infection in the use of banked allograft bone.. J Bone and Joint Surg (Am).

[CIT0032] Tsuruga E, Takita H, Itoh H, Wakisaka Y, Kuboki Y (1997). Pore size of porous hydroxyapatite as the cell-substratum controls BMP-induced osteogenesis.. J Biochem.

[CIT0033] Van Haaren EH, Smit TH, Phipps K, Wuisman PI, Blunn G, Heyligers IC (2005). Tricalcium-phosphate and hydroxyapatite bone-graft extender for use in impaction grafting revision surgery. An in vitro study on human femora.. J Bone Joint Surg (Br).

[CIT0034] Van Haaren EH, Heyligers IC, Alexander FG, Wuisman PI (2007). High rate of failure of impaction grafting in large acetabular defects.. J Bone Joint Surg (Br).

[CIT0035] Ware J, Kosinski M, Keller SD (1996). A 12-Item Short-Form Health Survey: construction of scales and preliminary tests of reliability and validity.. Medical Care.

